# Adaptation of short-term plasticity parameters via error-driven learning may explain the correlation between activity-dependent synaptic properties, connectivity motifs and target specificity

**DOI:** 10.3389/fncom.2014.00175

**Published:** 2015-01-29

**Authors:** Umberto Esposito, Michele Giugliano, Eleni Vasilaki

**Affiliations:** ^1^Department Computer Science, University of SheffieldSheffield, UK; ^2^Theoretical Neurobiology and Neuroengineering Laboratory, Department Biomedical Sciences, University of AntwerpAntwerp, Belgium; ^3^Laboratory of Neural Microcircuitry, Brain Mind Institute, Swiss Federal Institute of Technology of LausanneÉcole Polytechnique Fédérale de Lausanne, Switzerland; ^4^INSIGNEO Institute for in Silico Medicine, University of SheffieldSheffield, UK

**Keywords:** short-term plasticity, long-term plasticity, learning, rate code, motifs, target-specificity

## Abstract

The anatomical connectivity among neurons has been experimentally found to be largely non-random across brain areas. This means that certain connectivity motifs occur at a higher frequency than would be expected by chance. Of particular interest, short-term synaptic plasticity properties were found to colocalize with specific motifs: an over-expression of bidirectional motifs has been found in neuronal pairs where short-term facilitation dominates synaptic transmission among the neurons, whereas an over-expression of unidirectional motifs has been observed in neuronal pairs where short-term depression dominates. In previous work we found that, given a network with fixed short-term properties, the interaction between short- and long-term plasticity of synaptic transmission is sufficient for the emergence of specific motifs. Here, we introduce an error-driven learning mechanism for short-term plasticity that may explain how such observed correspondences develop from randomly initialized dynamic synapses. By allowing synapses to change their properties, neurons are able to adapt their own activity depending on an error signal. This results in more rich dynamics and also, provided that the learning mechanism is target-specific, leads to specialized groups of synapses projecting onto functionally different targets, qualitatively replicating the experimental results of Wang and collaborators.

## 1. Introduction

It is the current belief that experiences and memories are registered in long-term stable synaptic changes. Long-term plasticity, and in particular Hebbian learning or Spike-Timing-Dependent-Plasticity (STDP), is a form of unsupervised learning that captures correlations in the neuronal input. Hence, their involvement in, for instance, the development of receptive fields (e.g., Song et al., 2000; Clopath et al., 2010) or memory and associations is long-standing knowledge. However, the variety of different long-term plasticity rules (Markram et al., 2011), indicates that the precise synaptic prescriptions of long-term plasticity mechanisms remain unclear.

On the contrary, short-term plasticity (STP) is well-described (Varela et al., 1997; Markram et al., 1998b; Le Be' and Markram, 2006; Rinaldi et al., 2008; Testa-Silva et al., 2012; Costa et al., 2013; Romani et al., 2013) in the context of specific models (Tsodyks and Markram, 1997; Hennig, 2013; Rotman and Klyachko, 2013). Its role in neuronal computation is assumed to be related to temporal processing, see for instance (Natschläger et al., 2001) or the work by Carvalho and Buonomano (2011), where STP is demonstrated to enhance the discrimination ability of a single neuron (i.e., a tempotron, see Gütig and Sompolinsky, 2006), when presented with forward and reverse patterns. Synapses with STP are also optimal estimators of presynaptic membrane potentials (Pfister et al., 2010).

The investigation of the brain wiring diagram known as *connectomics* has recently made spectacular progress and generated excitement for its perspectives (Seung, 2009). Novel discoveries in molecular biology (Wickersham et al., 2007; Zhang et al., 2007; Lichtman et al., 2008), neuroanatomical methods (Denk and Horstmann, 2004; Chklovskii et al., 2010), electrophysiology (Song et al., 2005; Hai et al., 2010; Perin et al., 2011), and imaging (Friston, 2011; Minderer et al., 2012; Wedeen et al., 2012) have pushed forward the technological limits for ultimate access to neuronal connectivity. The comprehension of this level of organization of the brain (Kandell et al., 2008) is pivotal to understanding the richness of its high-level cognitive, computational and adaptive properties, as well as its dysfunctions.

At the microcircuit level (Binzegger and Douglas, 2004; Grillner et al., 2005; Silberberg et al., 2005; Douglas and Martin, 2007a,b), the non-random features of cortical connectivity have recently raised a lot of interest (Song et al., 2005; Perin et al., 2011). The occurrence of stereotypical connectivity motifs was experimentally demonstrated and, in some cases, accompanied by physiological information on neuronal and synaptic properties (Song et al., 2005; Wang et al., 2006; Silberberg and Markram, 2007; Perin et al., 2011), on activity-dependent short-term and long-term plasticity (Buonomano and Merzenich, 1998) and rewiring (Chklovskii et al., 2004; Le Be' and Markram, 2006). Recent experimental findings obtained in young ferret cortices (Wang et al., 2006) indicate that short-term facilitation and depression correlate to specific connectivity motifs: neurons connected by synapses exhibiting short-term facilitation form predominantly reciprocal (bidirectional) motifs; neurons connected by synapses exhibiting short-term depression form unidirectional motifs. Interestingly, the same overexpression of connectivity motifs has been observed in another brain area, i.e., the excitatory microcircuitry of the olfactory bulb (Pignatelli, 2009).

Earlier work by Vasilaki and Giugliano (2012, 2014) attempted to shed light on this correlation between STP and the observed wiring diagram configuration. They demonstrate that all facilitating or all depressing networks, upon receiving the same wave-like stimulation, give rise to the experimentally observed motifs: bidirectional for facilitating synapses and unidirectional for depressing synapses. This was explained both in the context of mean field analysis and microscopic simulations as a frequency-dependent effect. This is a simple consequence of the type of input (wave like) and the choice of the STDP triplet rule (Pfister and Gerstner, 2006). Differently from the classical pair rule, the triplet rule displays a frequency-dependent behavior, which can explain some experimental results (Sjöström et al., 2001): at low frequencies the rule reveals the classic STDP and, given a wave-like input, it results in unidirectional connectivity (Clopath et al., 2010; Vasilaki and Giugliano, 2014). At high frequencies, however, it reveals “classic Hebb” behavior: neurons that fire together, wire together. Hence, the low firing network develops unidirectional connectivity, while the high firing network develops bidirectional connectivity; for details see (Vasilaki and Giugliano, 2014). However, the observed synaptic development was not associated to any particular type of learning, but was explored as the emerging structure upon receiving a wave like input: what the network learned *per se* in that context was not clear.

With the present work we aim to complement and extend on Vasilaki and Giugliano (2012, 2014). We define a learning model for STP through which a population of neurons can modify its synapses in order to adapt its own activity and then fulfill a given time-varying task. The key idea comes from an optimization perspective: neurons that are able to modify their synapses, for instance making depressing synapses more and more depressing or even turning them into facilitating ones, would allow for much more flexibility and efficacy in signal transmission. A similar argument can be found in Markram et al. (1998a), whereas for earlier but different mechanisms of STP optimization or learning we redirect to Natschläger et al. (2001) and Carvalho and Buonomano (2011).

Then, we construct a typical inverted associative learning problem (Asaad et al., 1998; Fusi et al., 2007; Vasilaki et al., 2009b) where neurons have to learn to respond with high or low frequencies, when presented with the same wave-like input signal. We use this paradigm to show the potential of our model. In particular, not only do we provide an explanation for the correspondence motifs-synaptic properties within the context of learning both STP and STDP (triplet rule) but we also qualitatively capture, for instance, the heterogeneity in synaptic properties observed by Wang et al. (2006).

Moreover, having defined the learning model as a target-specific mechanism, we are able to obtain variability in the short-term profile of synapses innervating functionally different targets. Finally, we show that the learning model can be reduced to a minimal model where only the time constant of recovery from depression τ_*rec*_ needs to be learnt in order to obtain neurons firing at high or low frequency. Comparing this finding with the results from Carvalho and Buonomano (2011), we suggest that different parameters of the model describing STP might be related to different types of coding.

## 2. Materials and methods

### 2.1. Single neuron model

Each neuron is modeled as in Carvalho and Buonomano (2011): the sub-threshold dynamics of the electrical potential *V*_*i*_ of the generic neuron *i* are described by the equation:

(1) $$\frac{dV_{i}}{dt}=-g_{L}V_{i}+\sum_{j\;=\;1,\;j\;\neq \;i}^{N}g_{ij}\left(E_{rev}-V_{i}\right),$$

where *E*_*rev*_ = 30 mV is the reversal potential and *g*_*L*_ = 0.1 μS is the leak conductance - both quantities are equal and fixed for all neurons. {*g*_*ij*_}_*i,j* = 1, … *N*_ is the matrix of conductances and the generic element *g*_*ij*_ represents the conductance of the synapse going from neuron *j* to neuron *i*. Upon arrival of a presynaptic action potential elicited by neuron *j*, each of the conductances *g*_*ij*_ with *i* = 1, … *N*, *i* ≠ *j* increases by a quantity *w*_*ij*_, called effective synaptic efficacy, and decays exponentially back to zero with a fixed time constant τ_*g*_ = 10 ms, equal for all synapses:

(2) $$\frac{dg_{ij}}{dt}=-\frac{g_{ij}}{\tau_{g}}+\sum_{f}w_{ij}\delta \left(t-t_{j}^{f}\right),$$

where *t*^*f*^_*j*_ is the *f*-th spike emitted by neuron *j*. The effective synaptic efficacy depends on both presynaptic and postsynaptic factors:

(3) $$w_{ij}=r_{ij}u_{ij}A_{ij},$$

where *r*_*ij*_ and *u*_*ij*_ are the presynaptic variables representing depression and facilitation in the STP model (see Subsection 2.2) and *A*_*ij*_ is the postsynaptic variable for the maximum synaptic strength (or absolute efficacy), which represents the maximum synaptic response (see Subsection 2.7). If *V*_*i*_(*t*) ≥ 1 mV a spike is elicited by neuron *i* and *V*_*i*_(*t* + *dt*) is set to 0 for the next *t*_*ref*_ = 10 ms (refractory period).

### 2.2. STP model

Short-term synaptic plasticity is described at each synapse through the evolution of two variables, *r*_*ij*_ and *u*_*ij*_, representing the degree of depression and facilitation of the synapse connecting neuron *j* to neuron *i*. The time course of *r*_*ij*_ and *u*_*ij*_ is given by the following kinetic equations (Markram et al., 1998b; Maass and Markram, 2002):

(4) $$\frac{dr_{ij}}{dt}=\frac{1-r_{ij}}{\tau_{rec_{ij}}}-\sum_{j\;=\;1,\;j\;\neq \;i}^{N}\sum_{f}r_{ij}u_{ij}\delta \left(t-t_{j}^{f}\right)$$

(5) $$\frac{du_{ij}}{dt}=\frac{U_{ij}-u_{ij}}{\tau_{facil_{ij}}}+\sum_{j\;=\;1,\;j\;\neq \;i}^{N}\sum_{f}U_{ij}\left(1-u_{ij}\right)\delta \left(t-t_{j}^{f}\right).$$

*U*_*ij*_, τ_*rec*_*ij*__ and τ_*facil*_*ij*__ are the parameters of the model and they represent, respectively: fraction of resources used by the first action potential, time constant of recovery from depression and time constant of synaptic facilitation. A learning rule for STP has to allow changes to (at least one of) these parameters. At each synapse, the product of *r*_*ij*_ and *u*_*ij*_ determines the presynaptic efficacy.

### 2.3. STDP model

We use the triplet learning rule defined by Pfister and Gerstner (2006) with hard bounds: maximum weights can only vary in the interval [*A*_*min*_, *A*_*max*_]. In this model, each neuron has two presynaptic variables *m*^1^, *m*^2^ and two postsynaptic variables *o*^1^, *o*^2^. In the absence of any activity, these variables exponentially decay toward zero with different time constants:

(6) $$\begin{array}{l} \tau_{m_{1}}\frac{dm_{i}^{1}}{dt}=-m_{i}^{1}\;\tau_{m_{2}}\frac{dm_{i}^{2}}{dt}=-m_{i}^{2}\\ \;\tau_{o_{1}}\frac{do_{i}^{1}}{dt}=-o_{i}^{1}\;\tau_{o_{2}}\frac{do_{i}^{2}}{dt}=-o_{i}^{2} \end{array}$$

whereas when the neuron elicits a spike they increase by 1:

(7) $$m_{i}^{1}\to m_{i}^{1}+1\text{ ​​}m_{i}^{2}\to m_{i}^{2}+1\text{ ​​}o_{i}^{1}\to o_{i}^{1}+1\text{ ​​}o_{i}^{2}\to o_{i}^{2}+1.$$

Then, assuming that neuron *i* fires a spike, the STDP implementation of the triplet rule can be written as follows:

(8) $$\{\begin{array}{l} \Delta A_{ji}^{STDP}=-\gamma o_{j}^{1}\left(t\right)\left[A_{2}^{-}+A_{3}^{-}m_{i}^{2}\left(t-\epsilon \right)\right]\\ \Delta A_{ij}^{STDP}=+\gamma m_{j}^{1}\left(t\right)\left[A_{2}^{+}+A_{3}^{+}o_{i}^{2}\left(t-\epsilon \right)\right] \end{array}$$

where γ is the learning rate, ϵ is an infinitesimal time constant to ensure that the values of *m*^2^_*i*_ and *o*^2^_*i*_ used are the ones right before the update due to the spike of neuron *i*, and *A*_*ij*_ is the maximum strength of the connection from *j* to *i*. Values of STDP amplitudes are taken from Pfister and Gerstner (2006) and are listed in Table 1.

**Table 1:** **Parameters used in simulations**.

**Symbol**	**Description**	**Value**
*N*	Number of total neurons	{40, 80}
*N*_*in*_	Number of input neurons	{30, 60}
*N*_*out*_	Number of output neurons	10
*E*_*rev*_	Reversal potential	30 mV
*g*_*L*_	Decay constant of neuron potential	0.1 μS
τ_*g*_	Decay constant of synaptic conductances	10 ms
*V*_*thr*_	Threshold potential for spike emission	1 mV
*t*_*ref*_	Refractory period	10 ms
ν_*in*_	Input firing rate	10 Hz
ν_*targ*_	Output target firing rate	{2, 20} Hz
τ^*min*^_*facil*_	Facilitation time constant - minimum value	1 ms
τ^*max*^_*facil*_	Facilitation time constant - maximum value	000 ms
τ^*min*^_*rec*_	Depression time constant - minimum value	100 ms
τ^*max*^_*rec*_	Depression time constant - maximum value	900 ms
*U*^*min*^	Synaptic utilization - minimum value	0.05
*U*^*max*^	Synaptic utilization - maximum value	0.95
η	Fixed learning rate for *U*, τ_*rec*_, τ_*facil*_	0.1
*A*^+^_2_	Amplitude of maximum weights change - pair term in Long-Term Potentiation	4.6 × 10^−3^
*A*^+^_3_	Amplitude of maximum weights change - triplet term in Long-Term Potentiation	9.1 × 10^−3^
*A*^−^_2_	Amplitude of maximum weights change - pair term in Long-Term Depression	3.0 × 10^−3^
*A*^−^_3_	Amplitude of maximum weights change - triplet term in Long-Term Depression	7.5 × 10^−9^
τ_*m*_1__	Decay constant of presynaptic indicator *m*_1_	16.8 ms
τ_*m*_2__	Decay constant of presynaptic indicator *m*_2_	575 ms
τ_*o*_1__	Decay constant of postsynaptic indicator *o*_1_	33.7 ms
τ_*o*_2__	Decay constant of postsynaptic indicator *o*_2_	47 ms
*A*_*min*_	Lower bound for maximum synaptic weights	0.001
*A*_*max*_	Higher bound for maximum synaptic weights	1
γ	Learning rate for STDP and for STP-dependent Δ*w*	{1, 2}
*dt*	Discretization time step	1 ms

STDP parameters are as in the nearest-spike triplet-model, described in Pfister and Gerstner (2006).

In order to set *A*_*min*_ we note that if the maximum weights connecting the input neurons to a specific output neuron all collapse to zero in the low firing rate regime, then, in the subsequent high firing rate regime, inputs were not able to “wake up” this neuron: it remained almost silent all the time. To avoid this, we set *A*_*min*_ = 10^−3^. With such a small value we can still apply the symmetry measure (Esposito et al., 2014), which assumes *A*_*min*_ = 0, see Subsection 2.9, to evaluate the symmetry of the network.

### 2.4. Learning task

Neurons are divided into different populations, each of them is required to fire at one of the two target firing rates: 30 Hz (*high*) or 5 Hz (*low*). To allow the populations to reach their target rate, both short- and long-term plasticity parameters are adapted via error-driven learning (see Subsection 2.6) and, in addition, the maximum synaptic strength is shaped by the STDP triplet rule (see Subsection 2.3).

### 2.5. Input signal and input neurons

In all simulations, the input signal is delivered only to a subset of neurons in the network, which we call input neurons *N*_*in*_. Each of these neurons receives a pulse-like stimulus with a fixed frequency ν_*in*_ = 10 Hz, whose amplitude (2 mV) is chosen to always elicit an action potential in the corresponding input neuron. The stimulus delivery, however, is not synchronous across the input neurons, but it follows a *sequential protocol*: neurons are stimulated one after another with a fixed time delay *t*_*delay*_ and in a fixed order. We choose *t*_*delay*_ = (ν_*in*_ *N*_*in*_)^−1^ so that neurons that belong to input cyclically receive a stimulus. To further explain this, one may imagine labeling the neurons depending on the order they receive the stimulus, and therefore on the firing order, then have the firing pattern *N*_1_, *N*_2_, *N*_3_, …, *N*_*N*_*in*__, *N*_1_, *N*_2_, *N*_3_, …, *N*_*N*_*in*__, *N*_1_, …, with each pair of adjacent spikes being separated by a time interval of *t*_*delay*_. We can think of the *N*_*in*_ neurons as if they are organized in a ring and the stimulus as a cyclically traveling wave across this ring. To include the effect of noise, a random Gaussian variable with zero mean and standard deviation equal to 0.1 *t*_*delay*_ is added to the firing times. The magnitude of the standard deviation is such that there is no inversion in the firing order. With this construction, the stimulus delivered to input neurons can be thought as generated by an external (not explicitly simulated) population of neurons where each external neuron projects only onto one corresponding input neuron.

**Figure 1:**
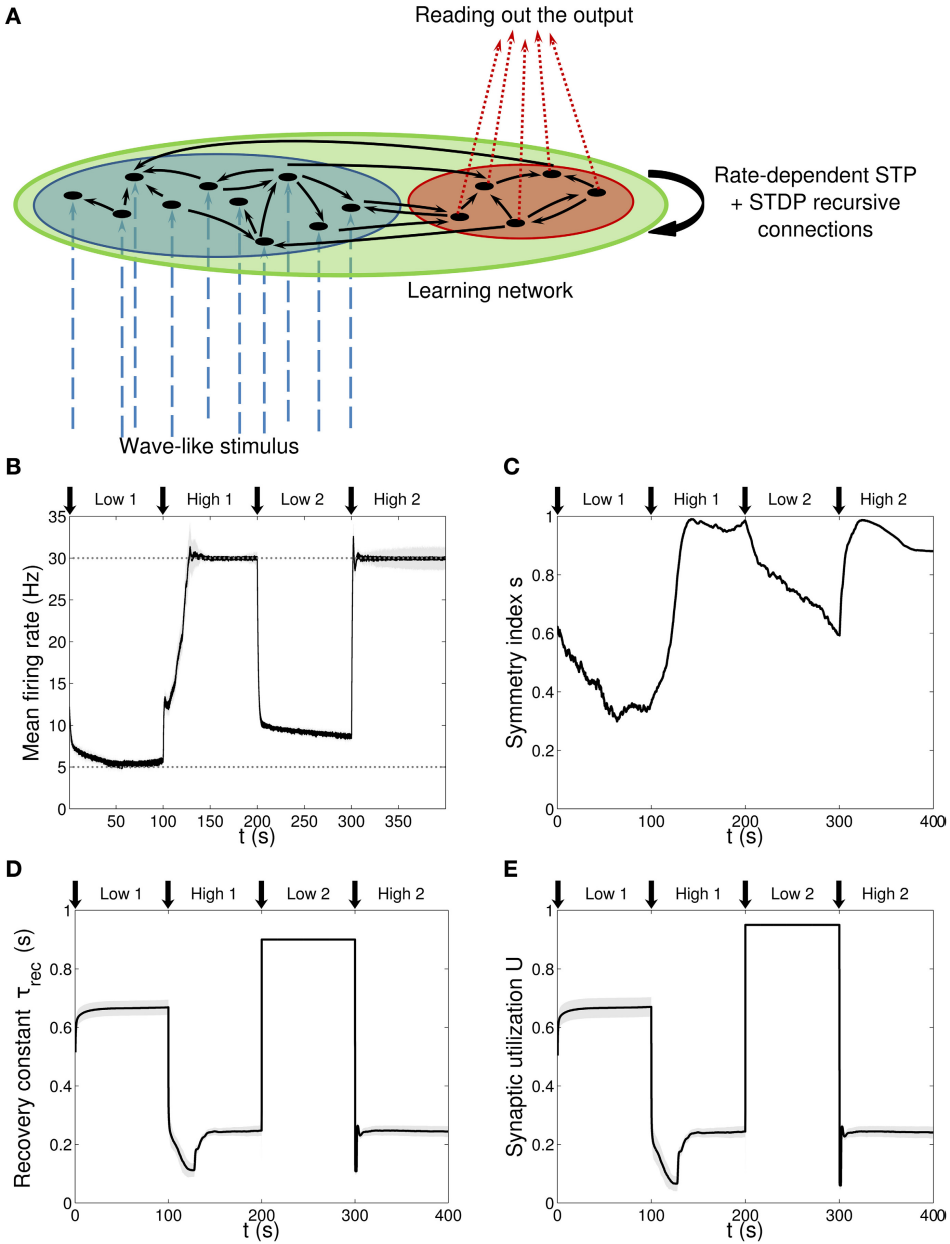
**Single population scenario: network architecture and activity, connectivity and STP parameters adaptation in the output population with (*U*, τ_*rec*_) learning scheme (*Part 1*). (A)** Architecture. The learning network (*green*) is divided into an input region (*blue*) and an output region (*red*). Connections (*black arrows*) are all-to-all and obey both Spike-Timing Dependent Plasticity and rate-dependent Short-Term Plasticity. Input neurons receive an external wave-like stimulus (*blue dashed arrows*). **(B)** Mean firing rate of the output population. *Shaded area* represents standard deviation, *horizontal dotted gray lines* show the two target firing rates (high = 30 Hz, low = 5 Hz) and *vertical black arrows* mark the onset of the four dynamic phases alternating the target according to the sequence low-high-low-high. **(C)** Symmetry measure applied on the connectivity of the output population. In accordance to the target, connectivity switches between unidirectionality (low values) and bidirectionality (high values). **(D,E)** Mean values of the recovery time constant τ_*rec*_ and synaptic utilization *U* for the synapses projecting onto the output neurons. We observe depression (high values) at low firing rates and facilitation (low values) at high firing rates.

Note that, by construction, in the absence of any other signal, the firing pattern of the input neurons reflects that of the stimulus. This means that the external signal implicitly fixes a level of minimum activation for the *N*_*in*_ neurons: their firing rate cannot be smaller than ν_*in*_. Due to this constraint, the input neurons, despite being free to change their parameters according to STP learning rules (see Subsection 2.6), are not totally free to regulate their firing activity, which may prevent them from effectively fulfilling the task. The rest of the neurons, instead, are totally free to adapt their activity and are called output neurons. For these reasons, we read out the interesting quantities only from output neurons (we refer to Results and to Figures 1A, **4A** for more details on the architecture).

### 2.6. Error-driven learning rule for STP

The task can be formulated as an optimization problem where neurons regulate their own activity in order to minimize the objective function defined as:

(9) $$E={\left(\frac{\nu_{targ}-\langle \nu \rangle }{\nu_{lim}}\right)}^{2},$$

where ν_*lim*_ is the maximum allowed frequency due to the refractory period (ν_*lim*_ = 1/*t*_*ref*_), ν_*targ*_ is the target firing rate and 〈ν〉 is the mean firing rate across a single population. To calculate firing rates of single neurons ν_*i*_ we use an exponential moving average with time constant τ_ν_ = 1 *s*:

(10) $$\tau_{\nu }\frac{d\nu_{i}}{dt}=-\nu_{i}+{\hat{\nu }}_{i}$$

where $\hat{\nu }$_*i*_ is the current firing rate, which basically reflects if neuron *i* has fired ($\hat{\nu }$_*i*_ = 1 Hz) or not ($\hat{\nu }$_*i*_ = 0 Hz). The population mean firing rate is therefore:

(11) $$\langle \nu \rangle =\frac{1}{N_{pop}}\sum_{i\;=\;1}^{N_{pop}}\nu_{i}$$

with *N*_*pop*_ being the size of the population.

By following a standard procedure, learning rules can be derived from Equation (9) by applying the gradient descent method (Hertz et al., 1991). Since the task is not based on single neurons but it involves an entire population, we use a mean-field approach for the derivation of the learning rules. Therefore, from now on in this section, we switch from the above single neuron notation to mean-field variables, by dropping the *ij* indices. It is worth noting that in our formulation the target is achieved not by directly acting on the firing rates, but by tuning the STP parameters, which in turn affects the firing itself. Therefore, 〈ν〉 = 〈ν〉 (*U*, τ_*rec*_, τ_*facil*_) and by using the chain rule we can formally write the following update rule for each parameter *p*:

(12) $$\begin{array}{l} \Delta p=-\eta_{p}\frac{\partial E}{\partial p}=-\eta_{p}\frac{\partial E}{\partial \langle \nu \rangle }\frac{\partial \langle \nu \rangle }{\partial p}=2\eta_{p}\frac{\nu_{targ}-\langle \nu \rangle }{\nu_{lim}^{2}}\frac{\partial \langle \nu \rangle }{\partial p},\\ \;p=U,\tau_{rec},\tau_{facil} \end{array}$$

where η_*p*_ is the learning rate, which in principle could be different for each parameter. The form of the function 〈ν〉 (*U*, τ_*rec*_, τ_*facil*_) can be derived with a semi-heuristic procedure, following (Vasilaki and Giugliano, 2014). Whenever possible, for the mean-field variables we use the same symbols as in Vasilaki and Giugliano (2014) for consistency. Thus, we introduce the mean-field variables *u*, *x*, *U*, and *A*, respectively describing facilitation, depression, synaptic utilization and maximum strength. We assume a threshold-linear gain function between input mean current *h* and output mean firing rate 〈ν〉 = *a* [(*h* − ϑ)]_+_, for some constants *a*, ϑ. We can then write the dynamic mean-field equations for a population of neurons recurrently connected by short-term synapses as follows (Chow et al., 2005):

(13) $$\{\begin{array}{l} \tau \dot{h}=-h+Aux\langle \nu \rangle +I_{ext}\\ \dot{x}=\frac{1-x}{\tau_{rec}}-ux\langle \nu \rangle \\ \dot{u}=\frac{U-u}{\tau_{facil}}-+U\left(1-u\right)\langle \nu \rangle \end{array}$$

where *I*_*ext*_ is the mean external current and τ is a decaying constant. By imposing equilibrium conditions, ḣ = ẋ = $\dot{u}$ = 0, and combining the resulting equations, we can finally write:

(14) $$\begin{array}{l} h=F\left({\langle \nu \rangle }_{h}\right)=\frac{AU\left({\langle \nu \rangle }^{-1}+\tau_{facil}\right)}{{\langle \nu \rangle }^{-2}+{\langle \nu \rangle }^{-1}U\tau_{facil}+{\langle \nu \rangle }^{-1}U\tau_{rec}+U\tau_{facil}\tau_{rec}}\\ \;+I_{ext} \end{array}$$

Now we observe that by taking the limit *h* → ∞ in *F*(〈ν〉_*h*_) we obtain an upper bound for the maximum allowed firing rate 〈ν〉 ≤ $\frac{A}{\tau_{rec}}$ + *I*_*ext*_ (for more details see Vasilaki and Giugliano, 2014). We can heuristically turn the above inequality into an equality:

(15) $$\langle \nu \rangle =\frac{A}{\tau_{rec}}+I_{ext}$$

so as by plugging Equation (15) into Equation (12) we can finally obtain an explicit form for the learning rule. In particular, since only one of the three parameters appears in Equation (15), we have a single rule for τ_*rec*_ only:

(16) $$\Delta \tau_{rec}=-2\eta_{\tau_{rec}}\left(\nu_{targ}-\langle \nu \rangle \right)\frac{A}{\nu_{lim}^{2}\tau_{rec}^{2}}$$

Then, according to the above derivation, the only parameter that needs to be learnt is τ_*rec*_. Here we adopt the view (Tsodyks and Markram, 1997; Markram et al., 1998b; Thomson, 2000; Chow et al., 2005) that facilitation/depression corresponds to small/large values of τ_*rec*_ and *U* as well. Therefore, assuming that they apparently play a similar role, we can heuristically take a similar dependence of 〈ν〉 upon *U*: 〈ν〉 = $\frac{A}{U}$ + *I*_*ext*_, which leads us to a similar learning rule:

(17) $$\Delta U=-2\eta_{U}\left(\nu_{targ}-\langle \nu \rangle \right)\frac{A}{\nu_{lim}^{2}U^{2}}$$

With the same heuristic argument we can also write down a relation involving τ_*facil*_. Indeed, it is well-know that facilitation/depression corresponds to large/small values of τ_*facil*_, so we can hypothesize a linear relation, also including the dependence on the maximum strength for similarity with the other parameters. Thus, 〈ν〉 ∝ *A*τ_*facil*_ + *I*_*ext*_, which gives the following learning rule:

(18) $$\Delta \tau_{facil}=2\eta_{\tau_{facil}}\left(\nu_{targ}-\langle \nu \rangle \right)\frac{A}{\nu_{lim}^{2}}$$

Finally, based on the fact that *A* turns out to appear in Equation (15), and supported by experimental results showing an interaction between STP and STDP (Markram et al., 1997; Sjöström et al., 2003), we can also introduce a STP-dependent learning rule for the maximum synaptic strength:

(19) $$\begin{array}{l} \Delta A^{STP}=-\eta_{A}\frac{\partial E}{\partial A}=-\eta_{A}\frac{\partial E}{\partial \langle \nu \rangle }\frac{\partial \langle \nu \rangle }{\partial A}\\ \;=2\eta_{A}\left(\nu_{targ}-\langle \nu \rangle \right)\frac{1}{\nu_{lim}^{2}\tau_{rec}}. \end{array}$$

This synaptic modification clearly does not substitute the traditional STDP, since the two rules come from different mechanisms. Rather, we assume they both contribute to maximum weights changes (see Subsection 2.7).

### 2.7. Single neuron learning framework: combining STDP and STP learning models

Equations (16–19) are mean field learning rules for the four parameters τ_*rec*_, *U*, τ_*facil*_, *A*. It is straightforward to turn them into single neuron online learning rules. From now on, we return to a single neuron notation. Similarly to STDP, we hypothesize that modifications of STP are triggered by postsynaptic events: every time neuron *i* elicits a spike, its current firing rate is updated as well as the mean population firing rate. Neuron *i* can therefore backwards regulate its incoming synapses, through the following set of equations:

(20) $$\;\Delta \tau_{rec_{ij}}=-2\eta_{\tau_{rec_{ij}}}\left(\nu_{targ}-\langle \nu \rangle \right)\frac{A_{ij}}{\nu_{lim}^{2}\tau_{rec_{ij}}^{2}}$$

(21) $$\;\Delta U_{ij}=-2\eta_{U_{ij}}\left(\nu_{targ}-\langle \nu \rangle \right)\frac{A_{ij}}{\nu_{lim}^{2}U_{ij}^{2}}$$

(22) $$\Delta \tau_{facil_{ij}}=2\eta_{\tau_{facil_{ij}}}\left(\nu_{targ}-\langle \nu \rangle \right)\frac{A_{ij}}{\nu_{lim}^{2}}$$

(23) $$\;\Delta A_{ij}^{STP}=2\eta_{A_{ij}}\left(\nu_{targ}-\langle \nu \rangle \right)\frac{1}{\nu_{lim}^{2}\tau_{rec_{ij}}}.$$

The firing event of the neuron *i* also triggers STDP, according with Equation (8). This contribution sums up with the above STP-dependent change, so as the total modification of the maximum synaptic strength is:

(24) $$A_{ij}\to A_{ij}+\Delta A_{ij}^{tot},\;\Delta A_{ij}^{tot}=\Delta A_{ij}^{STDP}+\Delta A_{ij}^{STP}.$$

Note that when we converted mean field population equations into single neuron equations we kept the population mean firing rate 〈ν〉, instead of turning it into the single rate ν_*i*_. This is because the task is defined at a population level. Learning rates of the three STP parameters are chosen to be equal and error-dependent:

(25) $$\eta_{p_{ij}}=\bar{\eta }{\left(1+\frac{\nu_{targ}-\langle \nu \rangle }{\nu_{lim}}\right)}^{2},\;p=U,\tau_{rec},\tau_{facil},$$

with η = 0.1. The learning rate for maximum synaptic strength, instead, is fixed in time and it is the same as the one used for STDP, η_*A*_*ij*__ ≡ γ.

Now we have four single neuron rules for the STP learning model, plus an equation for STDP and an equation for combining the different rules for the maximum synaptic strength. All these six rules together, Equations (8, 20–24) form a complete learning scheme for each neuron, which is implemented in our simulations. These rules are now local, since their computation takes place separately in each neuron, but receive a global signal encoding for the task performance error.

### 2.8. Investigation of different rule combinations

In the Results section we consider different learning mechanisms: in addition to STDP, that is crucial for the formation of motifs (Vasilaki and Giugliano, 2012, 2014), different combinations of the four STP rules are taken into account while the remaining parameters are kept fixed. At first we allow only two parameters to change: *(i)* τ_*rec*_, because Equation (15) implies that for high frequencies this is the only critical parameter for adapting the firing rate of the population, and *(ii) U*, since it was a key parameter adopted in the work in Carvalho and Buonomano (2011). Then, we introduce the STP-dependent rule on the maximum synaptic strength, Equation (23), with the view to observe a more stable learning process. Following this, we also include τ_*facil*_ in the learning scheme for a full parameter adaptation (*full model*) and finally we investigate the minimal number of parameters that needs to be adapted (*minimal model*), based on Equation (15). Looking for other parameter combinations might not be meaningful, as Equation (15) indicates the key parameters that are involved in changing the mean firing of the population.

### 2.9. Connectivity analysis

To reveal the type of connectivity in the output population, we use a symmetry index defined as a measure of the symmetry of the connectivity matrix *W* (Esposito et al., 2014):

(26) $$s=1-\frac{2}{N\left(N-1\right)-2M}\sum_{i\;=\;1}^{N}\sum_{j\;=\;i\;+\;1}^{N}\frac{\left|A_{ij}-A_{ji}\right|}{A_{ij}+A_{ji}}.$$

Here *M* is the number of instances where both *A*_*ij*_ and *A*_*ji*_ are zero, i.e., there is no connection between two neurons. Since in our case connections are bounded in the interval [10^−3^, 1], *M* = 0 all the time. Equation (26) is able to capture the presence of global non-random structures in a network, returning a value included in [0, 1]. Values of *s* close to 1 reflect the presence of a global bidirectional motif, whereas when *s* approaches 0, a unidirectional motif is emerging. Note that, in order to apply the measure Equation (26), we assume that the lower bound for connections is 0. However, the choice of a small value such as 10^−3^ does not affect the measure.

### 2.10. Data sharing

We provide the scripts that were used to construct the main figures of the paper in the ModelDB database, accession number:169242.

## 3. Results

### 3.1. Single population with a time-varying task: a continuum between facilitation and depression

First, we apply our learning model to a specific task demonstrating how synapses can change their behavior driven by an external feedback signal. The problem we study is simple: a population of neurons is presented with a stimulus and is required to produce a certain output as a response to that stimulus. Once the learning has been successful, for the same input signal the desirable output changes. In other words, neurons are trained to respond to a change in the associative paradigm (*inverted associative learning problem*), that can be due to, for instance, changes in the environmental conditions.

Let us give a concrete example of an inverted associative learning problem, taken from Asaad et al. (1998). In their work, the authors trained monkeys to associate visual stimuli (pictures) with delayed saccadic movements, left or right, with associations being reversed from time to time. Monkeys had to go beyond learning a single cue-response association: they are required to learn to associate, on alternate blocks, two cue objects with two different saccades. In other words, after having learned the relation {*object A, go right*}, and {*object B, go left*}, the associations were reversed such that now they needed to learn {*object A, go left*} and {*object B, go right*}.

Similar to the (Asaad et al., 1998) experiment, we assume a binary problem, i.e., environmental conditions can change only between two states, and we measure the neurons' activity in terms of firing rate. This means that neurons are initially asked to fire at some rate and, after learning this task, they are asked to fire at a different rate, while keeping the same input signal all the time. Thus, the problem we defined is a simpler version of the monkey experiment, with only a single input. In order to train the neurons on the current associative paradigm, an external global signal is required, that can be considered as an error signal (see Section Methods 2.6 and 2.7).

#### 3.1.1. Problem description and network architecture

We created a learning network of *N* = 40 conductance-based integrate-and-fire neurons (see Section Methods 2.1) all to all connected. Synaptic connections are modified by the STDP triplet rule (Pfister and Gerstner, 2006) and STP is implemented by using the Tsodyks and Markram model (TM model) described in Markram et al. (1998b); Maass and Markram (2002).

Figure 1A shows the network architecture, composed by two non-overlapping regions: a *blue* one with *N*_*in*_ = 30 neurons receiving the input signal and a *red* one with *N*_*out*_ = 10 neurons from which we read out the quantities of interest. Note that for clarity, only a few neurons (*black circles*) and connections (*black arrows*) are drawn. The network is therefore formed by two functionally distinct populations, with the input population delivering the stimulus to the output one. Recursive connections are present within each population and across populations, and they are all plastic, in the sense of both long-term and STP. We refer to this architecture as a first or single population scenario.

The input neurons are stimulated one after the other, following a *sequential protocol*, and approximately with the same rate, ν_*in*_ = 10 Hz. The amplitude of the stimulus is such that input neurons release a spike every time they receive an input (see Section Methods 2.5). This external source can be thought as an additional population of neurons, which we are not simulating here, where each “external” neuron is connected only with a corresponding neuron in the input population by means of a fixed feedforward connection (*blue dashed arrows*).

We hypothesize that the whole learning network (*green* region in Figure 1A) is presented with a sequence of two tasks while the stimulus pattern is kept fixed. The tasks are firing low (5 Hz) and firing high (30 Hz) and the sequence is *low-high-low-high*. Therefore, neurons have to repeatedly learn a new association and forget the previous one in a dynamic context divided in four phases of *t*_*ph*_ = 100 s. We refer to them as: low 1, high 1, low 2, high 2. As discussed at the beginning of this section, this picture is inspired by a typical inverted associative learning problem: considering the monkey experiment from Asaad et al. (1998) as a metaphor, our scenario provides a simplified version, where instead of having two different inputs, *object A* and *object B*, we have a single input. Indeed, we can think we are presenting the network with only *object A* and while doing this we switch the target association between the two states *go right* and *go left*, which correspond to our low and high firing rate targets. We call the desirable context-dependent target rate, ν_*targ*_. As described in Methods, the difference between ν_*targ*_ and the current firing rate of each population 〈ν〉 is the error signal that, according which our rate-dependent STP causes synapses to adapt their activity.

In all simulations, single neuron parameters {*U*, τ_*rec*_, τ_*facil*_, *w*}_*ij*_ are initially drawn from uniform distributions (for *i* ≠ *j*), respectively in [0.05, 0.95], [100, 900] ms, [1, 900] ms, [10^−3^, 1]. Synaptic variables are initialized at their equilibrium values, i.e., *r*_*ij*_ = 1 and *u*_*ij*_ = *U*_*ij*_. All the simulations in this subsection use γ = 1 for the high rate regime and γ = 2 for the low rate regime. Values of the parameters are listed in Table 1.

#### 3.1.2. Learning *U* and τ_*rec*_

**Figure 2:**
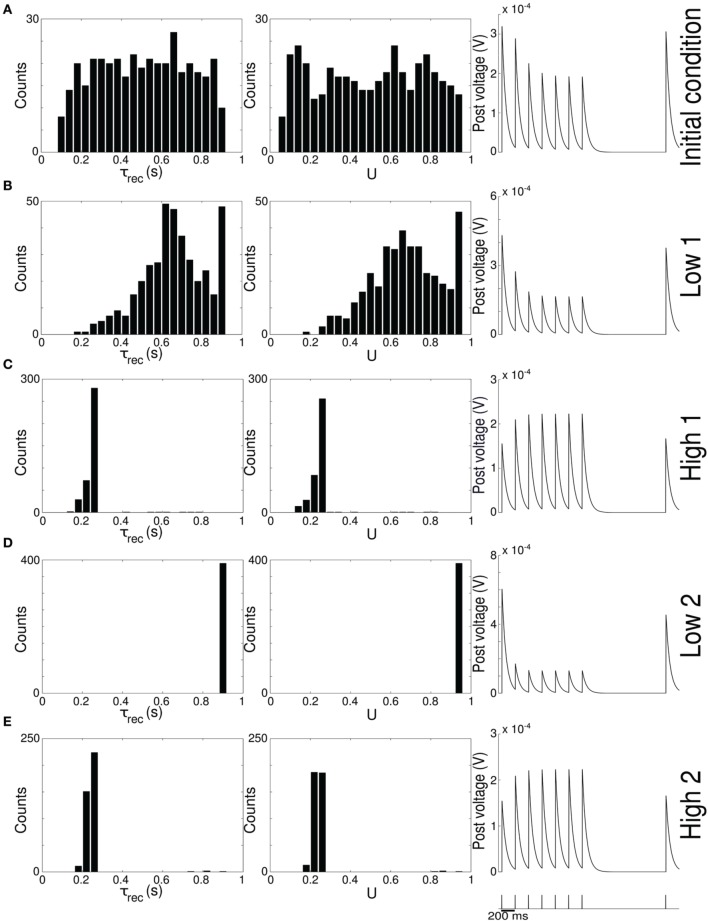
**Single population scenario: STP parameters distribution during adaptation with (*U*, τ_*rec*_) learning scheme (*Part 2*)**. Different phases of the dynamics are represented. **(A)** Initial (uniformly random) condition. **(B)** End of *low 1* phase (target rate is 5 Hz). **(C)** End of *high 1* phase (30 Hz). **(D)** End of *low 2* phase (5 Hz). **(E)** End of *high 2* phase (30 Hz). *Columns 1, 2* Histograms of recovery time constant and synaptic utilization of the synapses projecting onto the output neurons. Low values indicates facilitation whereas high values suggest depression. *Column 3* Single synapse traces obtained with the TM model by applying a 5 Hz stimulus. Synaptic parameters used are mean values obtained from the distributions drawn in **(A,B)**. Synapses display a clear alternation between depressing and facilitating behavior.

We initially studied the problem with a learning scheme involving *U* and τ_*rec*_ only, Equations (20, 21), so there is no additional change in maximum synaptic strengths due to STP. Indeed, due to Equation (15) and (Carvalho and Buonomano, 2011), we wanted to test the hypothesis that *U* and τ_*rec*_ are the only crucial parameters that need to be learnt for adapting the firing rate of a population. The results are displayed in Figures 1B–E, with *vertical black arrows* marking the beginning of each of the four phases, and in Figure 2.

Figure 1B shows the average firing rate of the *N*_*out*_ neurons, with *shaded area* being the standard deviation. Target firing rates are show with *gray dotted lines*. The adaptation to the new target is fast, except during the *low 2* phase, when we switch from high to low rate, where an initial fast decrease of the firing rate is followed by a much slower adaptation. Despite the fact that neurons do not reach the target rate during this phase, we observe a monotonically decreasing activity which would eventually stabilize at 5 Hz if we were allowing the simulation to run for longer. The reason for this double slope adaptation will be further discussed now.

Figure 1C shows the evolution of the symmetry index (see Section Methods 2.9). At the beginning, the value reflects the randomness in the connections (the mean value of *s* for a network with uniform random connections is indeed ≃ 0.614, see Esposito et al., 2014), whereas, as learning takes place, we observe the development of unidirectional (low values of *s*) and bidirectional (high values of *s*) motifs, depending on the set target. This can also be formalized by applying the *p*-value hypothesis test obtained by using mean and variance of *s* on a completely random network with uniform distribution of connections (Esposito et al., 2014). *P*-values are shown in Table 2. We, again, observe rather slow dynamics during the *low 2* phase that, within the fixed simulation time, prevent the system from reaching a clear connectivity configuration. However, the trend of *s* clearly shows that the connectivity within the output population is approaching unidirectionality.

**Table 2:** **Symmetry measure and *p*-value for the single population scenario with {τ_*rec*_, *U*} and {τ_*rec*_, *U*, *A*} learning schemes**.

**Phase**	**(τ_*rec*_, *U*) scheme**		**(τ_*rec*_, *U*, *w*) scheme**	
	***s***	***p*-value**	***s***	***p*-value**
*Low 1* (0-500 *s*)	0.36	1.82 × 10^−9^	0.28	1.13 × 10^−15^
*High 1* (500-1000 *s*)	0.98	5.47 × 10^−19^	0.99	8.73 × 10^−20^
*Low 2* (1000-1500 *s*)	0.59	6.47 × 10^−1^	0.41	2.19 × 10^−6^
*High 2* (1500-2000 *s*)	0.88	1.50 × 10^−10^	0.82	3.63 × 10^−7^

Column 1: the four phases of dynamics with the corresponding simulation time. *Columns 2,3*: symmetry measure and *p*-value for the adaptation of {τ_*rec*_, *U*}. Values are computed at end of each phase and by considering output neurons only. *Columns 4,5* same as columns 2 and 3 except for the adaptation of {τ_*rec*_, *U*, *A*}.

Figures 1D,E depicts the time course of the recovery time constant τ_*rec*_ and synaptic utilization *U* averaged across the output neurons, with *shaded area* representing standard deviation. Both parameters oscillate between high values, which correspond to depressing behavior, and low values, that indicate facilitation. Note that the dynamics of τ_*rec*_ and *U* is fast in all phases, the third included. This is not surprising since STP is a fast process and leads to fast adaptation of its parameters. As a result, neurons' response to a change in the target rate takes place in a short time. However, during the *low 2* phase, synaptic parameters saturate before the neurons could fulfill the task, with STDP being the only remaining mechanism through which the output population can regulate its own activity. This results in a much slower decrease toward the target rate for two reasons: *(i)* STDP by itself acts on much longer time scales, *(ii)* switching from high to low rate is the most challenging part of the entire sequence of tasks due to the saturation of the maximum weights in the previous *high 1* phase, which slows down the process even further.

Figure 2 provides additional evidence of the alternation between the two different synaptic behaviors. Plots are organized in five rows, with each row displaying information from a different phase of the simulation. Panel A shows the initial uniform condition, panel B the end of *low 1* phase, etc. For each stage, we draw the histograms of recovery time constant (*Column 1*) and synaptic utilization (*Column 2*). According to the narrow standard deviation observed in Figures 1D,E, distributions peak around extreme values, reflecting two different, synaptic behaviors. *Column 3* in Figure 2, displaying the single synapse traces obtained with a TM model, demonstrates the corresponding behaviors: at the end of the phases where neurons are required to fire low we observe a typical depressing response, whereas at the end of the high rate regimes synapses show a typical facilitating trace. To generate the traces, we used a 5 Hz signal to stimulate a synapse with a parameters given by the mean values obtained from the corresponding histograms. Note that the synaptic trace for the initial condition, i.e., before learning shapes the parameters, already shows depression, which explains why the distributions of τ_*rec*_ and *U* at the end of the *low 1* phase are much broader than in the following phases.

Altogether, the four panels **(B–E)** in Figure 1 and the five panels **(A–E)** in Figure 2 show that the properties and activity of the output population oscillate between two states and that the desirable structure is formed depending on the target rate. In particular, we observe that neurons that fire at low frequency turn their synaptic properties into depressing and the connections formed are mostly unidirectional. On the other hand, when the target rate is set at a high frequency, neurons develop facilitating synapses and bidirectional connections.

#### 3.1.3. STP-dependent modification of *A* enhances performance

Given the speed convergence issue in the *low 1* phase, we introduced an additional learning mechanism, i.e., the STP-dependent rule for *A*, Equations (23, 24). Indeed, this mechanism provides an additional way, besides the STDP, for regulating the long-term plastic synapses. In all the other aspects, the model remains as above.

**Figure 3:**
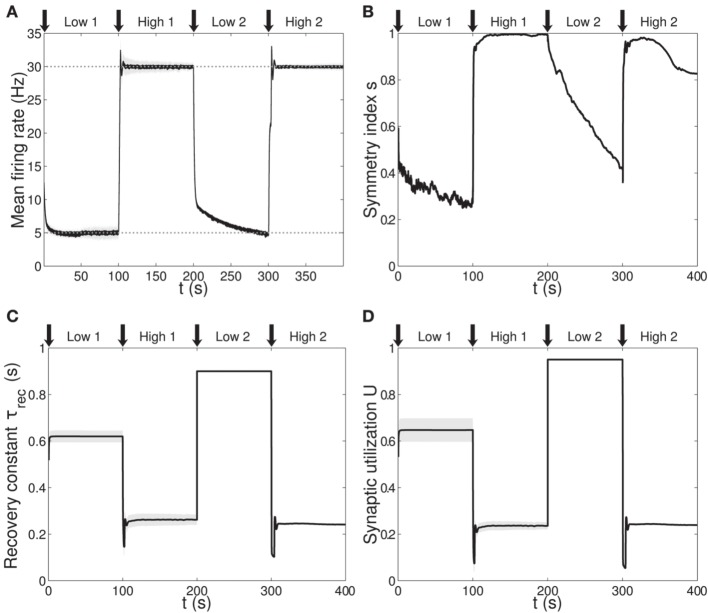
**Single population scenario: activity, connectivity and STP parameter adaptation in the output population with (*U*, τ_*rec*_, *A*) learning scheme. (A)** Mean firing rate of the output population. *Shaded area* represents standard deviation, *horizontal dotted gray lines* show the two target firing rates (high = 30 Hz, low = 5 Hz) and *vertical black arrows* mark the onset of the four dynamic phases alternating the targets in the sequence low-high-low-high. **(B)** Symmetry measure applied on the connectivity of the output population. In accordance with the target rate, connectivity switches between unidirectionality (low values) and bidirectionality (high values). **(C,D)** Mean values of recovery time constant τ_*rec*_ and synaptic utilization *U* for the synapses projecting onto the output neurons. We observe depression (high values) at low firing rates and facilitation (low values) at high firing rates. Compared to Figure 1, we observe an improvement in the overall performance due to the inclusion of the STP-dependent modification of *A*.

Figure 3 shows simulation results, with panels A-D depicting the same quantities as panels B-E in Figure 1 (symbols as before). A direct panel-by-panel comparison shows that the results are very similar, meaning that with this new learning configuration the output population also learns to adapt its synaptic properties in order to fulfill the current task, with subsequent motifs formation. As expected, due to the additional leaning rule for *A*, the dynamics are faster: in particular, during the *low 1* phase, neurons reach the target rate within the simulation time, and the value of the symmetry measure is much lower than before, confirming the formation of a unidirectional motif; compare with Figures 1C, 3B and see Table 2. Note that the adaptation of the STP parameters is also faster, as they depend on the current value of the maximum synaptic strength. Thus, the STP-dependent modification of *A* improves the overall performance and introduces an interesting link between STP and STDP.

### 3.2. Two populations with a different task: synaptic differentiation

Now we consider a different scenario, which we refer to as the second or double population scenario. The two tasks associated with low and high targets are now simultaneously active and must be learnt by different populations, interacting via lateral connections and receiving the same stimulus source. Reasons are multiple: we want to investigate if our model allows to contemporary encode both associative paradigms, without the need of forgetting one of the two. In addition, we want to study the possibility that target-specific STP emerges as a consequence of the target-dependent learning rules we chose for our model. In particular, we want to test whether our model is able to reproduce existing experimental data, specifically that appearing in Table 1 from the paper by Wang et al. (2006).

#### 3.2.1. Network architecture

**Figure 4:**
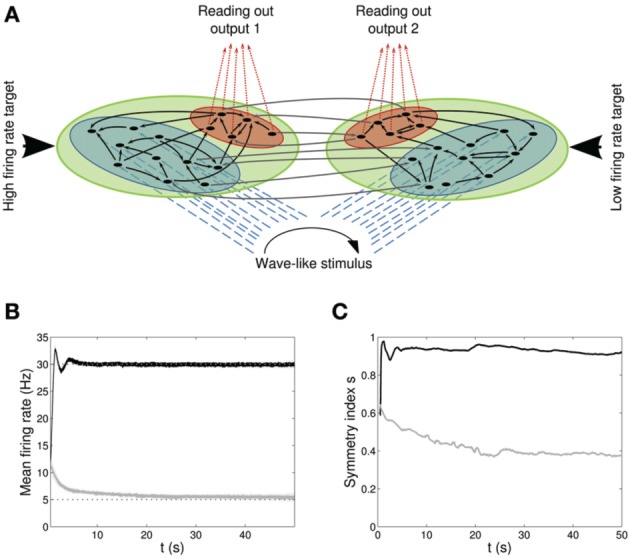
**Double population scenario: network architecture, activity and connectivity of the output populations with full (*U*, τ_*rec*_, τ_*facil*_, *A*) learning scheme (*Part 1*). (A)** Architecture. The previous network is doubled so that there are now four populations: two input regions (*blue*) and two output regions ℘^*out*^_1_, ℘^*out*^_2_ (*red*). The four populations are organized in two branches, one required to fire at high rates (30 Hz) and the second at low rates (5 Hz). Within each branch connections are all to all (*black arrows*) whereas initially weak connections (*gray arrows*) are present between the two output populations and between the two input populations. Input neurons receive a wave-like stimulus from outside (*blue dashed arrows*). All synapses obey both Spike-Timing Dependent Plasticity and rate-dependent Short-Term Plasticity. **(B)** Mean firing rate of the output populations, *black line* for ℘^*out*^_1_, and *gray line* for ℘^*out*^_2_. *Shaded area* represents standard deviation and *horizontal dotted gray lines* show the two target firing rates (30 Hz for ℘^*out*^_1_, 5 Hz for ℘^*out*^_2_). **(C)** Symmetry measure applied on the connectivity of the output population. Color legend as in **(B)**. Connectivity evolves differently in the two populations, leading to a bidirectional motif in ℘^*out*^_1_ and to a unidirectional motif in ℘^*out*^_2_.

The new configuration is depicted in Figure 4A and it is obtained by mirroring the structure of the first scenario and by adding recursive connections between functionally homologous populations. This led to a network of *N* = 80 conductance-based integrate-and-fire neurons, organized in two distinct branches of 40 neurons each, with the first branch required to fire at a high rate (ν = 30 Hz) and the second branch at a low rate (ν = 5 Hz). Both targets remain fixed throughout the entire simulation. Each branch is a replication of the architecture we used previously, i.e., it is formed by an input and an output population recursively connected. Thus, the network is formed by four functionally different populations: ℘^*in*^_1_, ℘^*in*^_2_, ℘^*out*^_1_, ℘^*out*^_2_, with obvious meaning of symbols. Input populations in both branches receive the stimulus from the same source: a single wave-like signal is delivered to the *N*_*input*_ = 60 neurons with ν = 10 Hz, stimulating one neuron per time (see Section Methods 2.5), first the neurons in ℘^*in*^_1_ and then the neurons in ℘^*in*^_2_. All connections are plastic following the STDP triplet rule and TM model for STP.

Lateral connections are present between the inputs ℘^*in*^_1_, ℘^*in*^_2_ and between the outputs ℘^*out*^_1_, ℘^*out*^_2_. To stress that they are functionally different, we drew their initial values from a uniform distribution in [10^−3^, 10^−1^], but, during the evolution, synapses are allowed to grow up to *A*_*max*_ = 1 as any other synapse. Furthermore, cross connections between different output and input populations, i.e., between ℘^*in*^_1_, ℘^*out*^_2_ and between ℘^*out*^_1_, ℘^*in*^_2_ are absent. The rest of the connections - within each population and across populations belonging to the same branch - are drawn from a uniform distribution in [10^−3^, 1] and they are not allowed to exceed this interval during the simulation. STP variables are initialized as in the single population scenario and in all the simulations presented in this subsection we used γ = 2 as the learning rate.

#### 3.2.2. Full model: adaptation of *U*, τ_*rec*_, τ_*facil*_ and *A*

**Figure 5:**
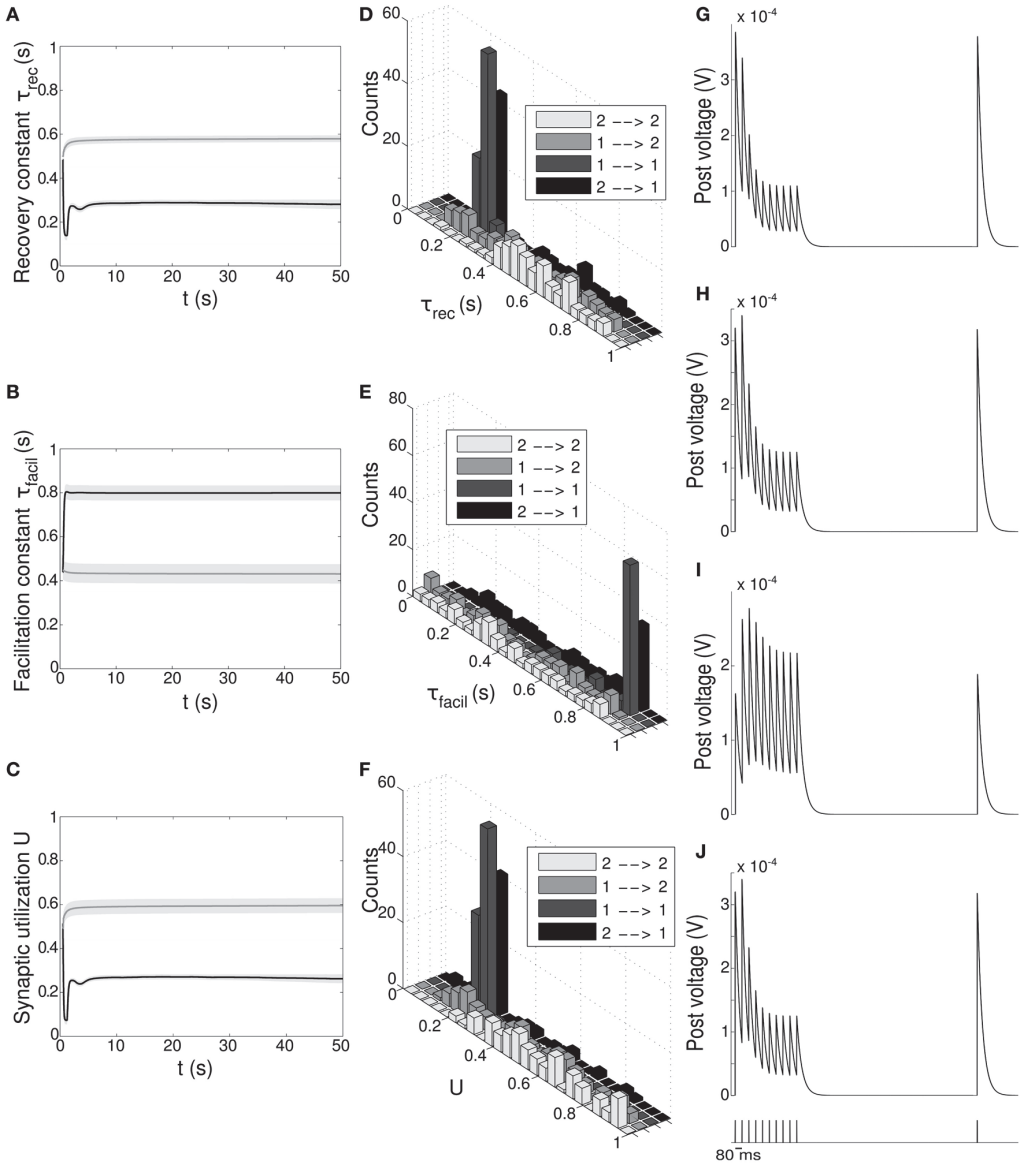
**Double population scenario: STP parameters adaptation and final distribution for the output populations with full (*U*, τ_*rec*_, τ_*facil*_, *A*) learning scheme (*Part 1*). (A–C)** Mean values of recovery time constant τ_*rec*_, facilitation time constant τ_*facil*_ and synaptic utilization *U*. *Black lines* represent mean values across the synapses projecting onto output population 1 from both output populations, ℘^*out*^_1_ ∪ ℘^*out*^_2_ → ℘^*out*^_1_, whereas *gray lines* describe the synapses projecting onto output population 2 from both output populations, ℘^*out*^_1_ ∪ ℘^*out*^_2_ → ℘^*out*^_2_. *Shaded areas* show standard deviation. We observe that the two populations develop different synaptic types, facilitating for ℘^*out*^_1_ and depressing for ℘^*out*^_2_. **(D–F)** Corresponding histograms of the three synaptic parameters at the end of the simulation. For each of them we show four different groups of values, mapping qualitatively to the four subtypes identified by Wang et al. (2006), see Table 3. *Light gray:* ℘^*out*^_2_ → ℘^*out*^_2_ (E2a). *Medium gray:* ℘^*out*^_1_ → ℘^*out*^_2_ (E2b). *Dark gray:* ℘^*out*^_1_ → ℘^*out*^_1_ (E1a). *Black:* ℘^*out*^_2_ → ℘^*out*^_1_ (E1b). **(G–J)** Single synapse traces obtained with the TM model by using a 12 Hz stimulus. Each panel represents a different subtype of synapses. **(G)** ℘^*out*^_2_ → ℘^*out*^_2_. **(H)** ℘^*out*^_1_ → ℘^*out*^_2_. **(I)** ℘^*out*^_1_ → ℘^*out*^_1_. **(J)** ℘^*out*^_2_ → ℘^*out*^_1_. Synaptic parameters used are the mean values obtained from the distributions drawn in **(D–F)**. A comparison with (Wang et al., 2006) on the basis of the traces only shows that we are able to identify three of the four subtypes.

We begin by studying the behavior of the *full model*: all four parameters are modified by our rate-dependent STP, Equations (20–24). Taking into account the modifications of all three STP parameters allows us to make a direct comparison with (Wang et al., 2006). Results are displayed in Figures 4B–C and in Figure 5.

Figures 4B,C shows the time course of the mean firing rate and symmetry index in both output populations, *black lines* for ℘^*out*^_1_ and *light gray lines* for ℘^*out*^_2_. *Shaded areas* and *dark gray dotted lines* represent standard deviation and target firing rates. Both populations ℘^*out*^_1_, ℘^*out*^_2_ approach the target rate while developing specific connectivity: as expected, a bidirectional motif emerges in the population firing at the high rate whereas the population firing at the low rate develops mostly unidirectional connections.

Figures 5A–C shows the time evolution of the three parameters of the TM model: *black lines* and *gray lines* represent the mean value of the synapses projecting from the two output populations ℘^*out*^_1_ ∪ ℘^*out*^_2_, respectively onto ℘^*out*^_1_ and ℘^*out*^_2_. Shaded area is the standard deviation. As expected from the previous simulation, we observe that the two populations develop different synaptic types: high values of τ_*facil*_ and low values of τ_*rec*_ and *U*, as observed in the population firing at the high rate, suggest a facilitating behavior, whereas values as the one observed in ℘^*out*^_2_, characterize depressing synapses. Mean values at the end of the simulation are reported in Table 3 *rows 1,4*. These results show that our model develops target-specific STP and results in good agreement with the data in Wang et al. (2006). Indeed, although single values are not identical, the qualitative synaptic behavior is represented: recalling the notation used in Wang et al. (2006), two main types of synapses are present. The group projecting from ℘^*out*^_1_ ∪ ℘^*out*^_2_ onto ℘^*out*^_1_ can be mapped onto the type *E*1 and the group projecting from ℘^*out*^_1_ ∪ ℘^*out*^_2_ onto ℘^*out*^_2_ that can be mapped onto the type *E*2.

**Table 3:** **Types and subtypes of excitatory synapses between the two output populations in the full model {τ_*rec*_, *U*, τ_*facil*_, *A*}**.

**Synaptic groups**	**τ_rec_(ms)**	**τ_*facil*_(*ms*)**	***U***	**τ_*rec*_/τ_*facil*_**	**τ_rec_/τ_facil_ as in Wang**	**Wang's subtypes**
℘^*out*^_1_ ∪ ℘^*out*^_2_ → ℘^*out*^_1_	310 ± 11	733 ± 17	0.27 ± 0.01	0.42	0.38	E1
℘^*out*^_1_ → ℘^*out*^_1_	260 ± 5	833 ± 13	0.25 ± 0.01	0.31	0.34	E1a
℘^*out*^_2_ → ℘^*out*^_1_	356 ± 19	643 ± 27	0.29 ± 0.01	0.55	0.43	E1b
℘^*out*^_2_ ∪ ℘^*out*^_1_ → ℘^*out*^_2_	550 ± 14	440 ± 19	0.55 ± 0.02	1.25	39.47	E2
℘^*out*^_2_ → ℘^*out*^_2_	595 ± 16	436 ± 26	0.61 ± 0.02	1.36	76.88	E2a
℘^*out*^_1_ → ℘^*out*^_2_	510 ± 23	443 ± 28	0.50 ± 0.03	1.15	25.55	E2b

*Column 1: synaptic groups. For instance ℘^out^_1_ ∪ ℘^out^_2_ → ℘^out^_1_ includes all synapses from both output populations, ℘^out^_1_ and ℘^out^_2_, to the output population firing high, ℘^out^_1_. Columns 2,3,4: mean values of STP parameters τ_rec_, τ_facil_, U. As in Wang et al. (2006), we provide the results in the form mean ± s.m.e.. Column 5: ratio between the two time constants, τ_rec_/τ_facil_, in our simulation. Column 6: for a direct comparison, we provide the values of τ_rec_/τ_facil_ as in Wang et al. (2006). Column 7: mapping of our subtypes onto Wang's subtypes*.

Following Wang et al. (2006), we can also refine our classification, introducing a further distinction within each class. With this purpose, we show in Figures 5D–F the distributions of τ_*rec*_, τ_*facil*_ and *U* at the end of the simulation within the entire output population ℘^*out*^_1_ ∪ ℘^*out*^_2_. For each histogram, data have been divided into four groups, representing the four different subtypes: ℘^*out*^_2_ to ℘^*out*^_2_ with *light gray*, ℘^*out*^_1_ to ℘^*out*^_2_ with *medium gray*, ℘^*out*^_1_ to ℘^*out*^_1_ with *dark gray*, ℘^*out*^_2_ to ℘^*out*^_1_ with *black*. While the distinction between the two synaptic types mapping onto *E*1 and *E*2 is evident, the difference between two subtypes in the same type cannot be easily seen. However, by looking at the mean values of synaptic parameters in Table 3 *rows 2, 3, 5, 6* and in particular the ratio τ_*rec*_/τ_*facil*_ in Table 3 *column 5*, the distinction into four subtypes becomes more clear. As reported in *column 7* of Table 3, we can map the synaptic subtypes as follows: *E*1*a* corresponds to the group ℘^*out*^_1_ → ℘^*out*^_1_, *E*1*b* to ℘^*out*^_2_ → ℘^*out*^_1_, *E*2*a* to ℘^*out*^_2_ → ℘^*out*^_2_ and *E*2*b* to ℘^*out*^_1_ → ℘^*out*^_2_.

Finally, similarly to Figure 2, in Figures 5G–J we show single synapse traces for each subtype. We observe that, except for the last trace, different groups effectively show a distinctive response to the same stimulus (12 Hz) and the traces reproduce the ones of the corresponding subtypes in Wang et al. (2006).

Although in Figures 5D–F we present four different histograms for each parameter, we can reason on the overall distribution within the entire output population ℘^*out*^_1_ ∪ ℘^*out*^_2_ as the sample size is the same in all histograms. We can therefore observe that the distribution of τ_*rec*_ closely matches that in Wang et al. (2006), whereas the distribution of *U* reproduces the peak at around 0.25 but is less broad. On the other hand, the distribution of τ_*facil*_ is rather different, being totally shifted toward facilitating values in our case. This may be due to the fact that *U* is much more peaked around low values. We decided then to discard τ_*facil*_ from the learning scheme and run a simulation where only *U*, τ_*rec*_ and *A* are learnt, as we did for the single population scenario in subsection 3.1.3. We observed that the behavior of the output populations and all the results remain unchanged. We provide an explanation for this in the Discussion.

#### 3.2.3. A minimal model for rate-dependent STP: adaptation of τ_*rec*_ and *A*

Finally, we study the *minimal model*: a model that suffices to obtain the desired behaviors by adapting as few parameters as possible. The choice of the parameters to be learnt is naturally suggested by the form of the objective function Equation (9): τ_*rec*_ and *A*. Interestingly, this minimal model preserves two key features: *(i)* both a presynaptic parameter, τ_*rec*_, and a postsynaptic parameter, *A*, participate in learning, *(ii)* STP and STDP are linked to each other through the STP-dependent modification of *A*.

**Figure 6:**
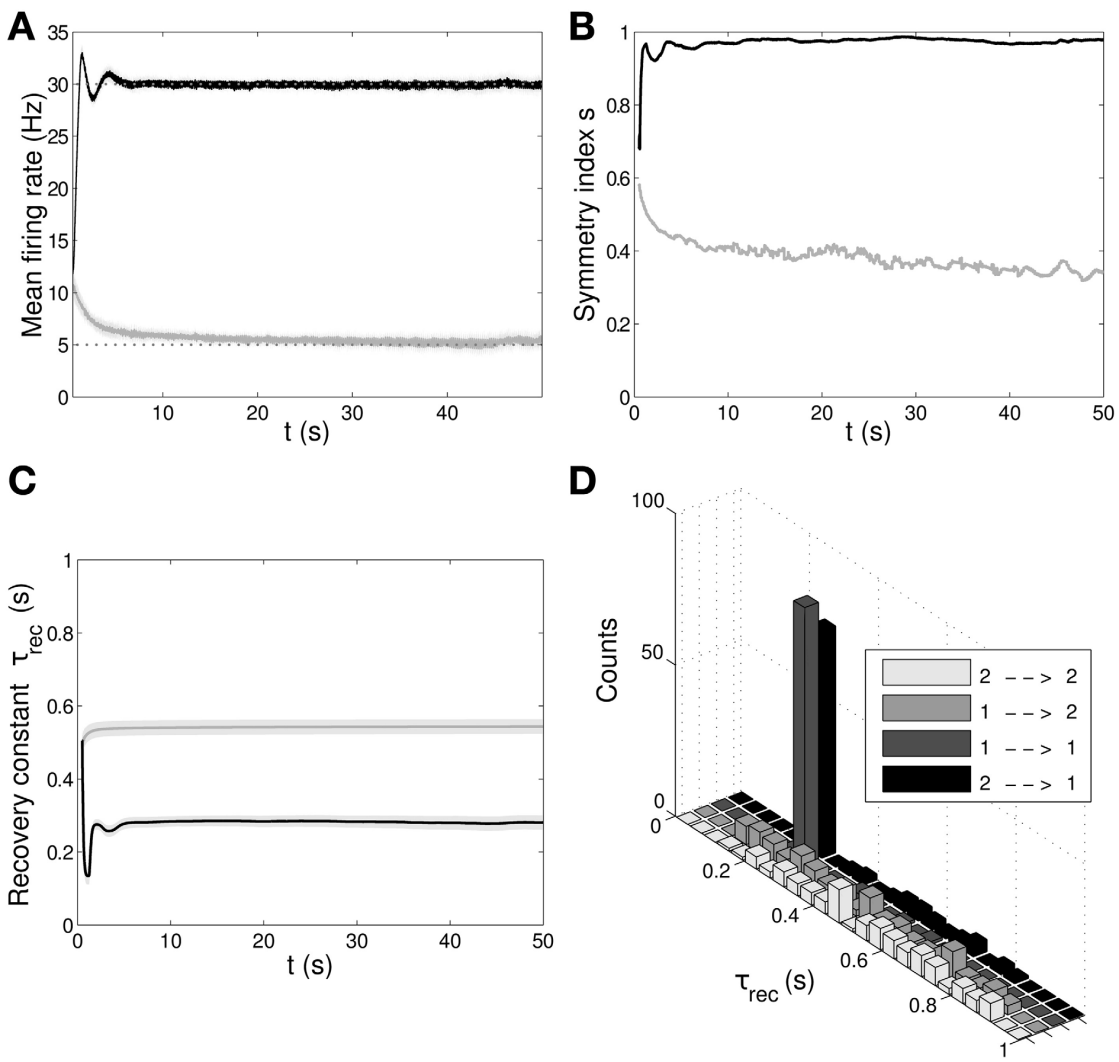
**Double population scenario: learning in the output populations with minimal (τ_*rec*_, *A*) model. (A)** Mean firing rate of the output populations, *black line* for ℘^*out*^_1_ and *gray line* for ℘^*out*^_2_. *Shaded area* represents standard deviation and *horizontal dotted gray lines* show the two target firing rates (30 Hz for ℘^*out*^_1_, 5 Hz for ℘^*out*^_2_). **(B)** Symmetry measure applied on the connectivity of the output population. Color legend as in **(B)**. Connectivity evolves differently in the two populations, leading to a bidirectional motif in ℘^*out*^_1_ and to a unidirectional motif in ℘^*out*^_2_. **(C)** Mean value of recovery time constant τ_*rec*_. *Black line:* ℘^*out*^_1_ ∪ ℘^*out*^_2_ → ℘^*out*^_1_. *Gray line:* ℘^*out*^_1_ ∪ ℘^*out*^_2_ → ℘^*out*^_2_. We observe that the two populations develop different type of synapses, facilitating for ℘^*out*^_1_ and depressing for ℘^*out*^_2_. **(D)** Corresponding histograms of the recovery time constant at the end of the simulation. *Light gray*: ℘^*out*^_2_ → ℘^*out*^_2_, *medium gray*: ℘^*out*^_1_ → ℘^*out*^_2_, *dark gray*: ℘^*out*^_1_ → ℘^*out*^_1_, *black*: ℘^*out*^_2_ → ℘^*out*^_1_. The panels show that the achievement of the tasks and the differentiation of the synapses is still possible with this minimal model.

In Figure 6 we show the results of the minimal model: from A to D, respectively: mean output firing rates, symmetry index, τ_*rec*_ evolution and τ_*rec*_ distribution in the four groups of synapses. By comparing these panels with the ones from the full model simulation, we observe that output populations still efficiently fulfill the task while developing the expected connectivity motifs. Also, in Table 4 we report the mean values of τ_*rec*_ for the four groups of synapses that we identified with the full model: there is still a clear distinction between them. We can therefore conclude that this minimal model is sufficient for qualitatively reproducing the main two types and also the subtypes of Wang et al. (2006).

**Table 4:** **Types and subtypes of excitatory synapses between the two output populations in the minimal model (τ_*rec*_, *A*)**.

**Synaptic groups**	**τ_rec_(ms)**
℘^*out*^_1_ ∪ ℘^*out*^_2_ → ℘^*out*^_1_	300 ± 9
℘^*out*^_1_ → ℘^*out*^_1_	267 ± 6
℘^*out*^_2_ → ℘^*out*^_1_	327 ± 15
℘^*out*^_2_ ∪ ℘^*out*^_1_ → ℘^*out*^_2_	524 ± 16
℘^*out*^_1_ → ℘^*out*^_2_	486 ± 23
℘^*out*^_2_ → ℘^*out*^_2_	567 ± 22

Symbols are as in Table 3. Similar to Wang et al. (2006), we provide the results in the form *mean* ± s.m.e.

## 4. Discussion

It is well-known that synapses are activity-dependent connections through which neurons propagate information. STP is a mechanism that describes these phenomena in short time scales and introduces two typical synaptic behaviors: depression and facilitation. Contrary to long-lasting modifications of maximum synaptic strengths, for example STDP, existing models of STP do not rely on any learning mechanisms, apart from very few exceptions; see for instance (Carvalho and Buonomano, 2011). Motivated by their work, it is our belief that more efficient dynamics would be possible if synapses were allowed to change their short-term behavior by tuning their own parameters, depending on one or more external controlling factors, for example, their current task. Typically, one asks which is the firing regime for which a certain type of synapse performs better (Barak and Tsodyks, 2007), whereas we are looking at the picture from a reverse perspective: we want to obtain some frequency regime, which is the most efficient way to do it from a synaptic point of view? A similar concept can be found in Natschläger et al. (2001), where the authors trained a network with a temporal structured target signal, using optimization techniques.

In our work, we developed a learning scheme for STP, and we obtained, with a semi-rigorous argument, a learning rule for only one of the three parameters of the TM model, τ_*rec*_. Based on specific experimental results (Tsodyks and Markram, 1997; Markram et al., 1998b; Thomson, 2000) and data fitting (Chow et al., 2005), we used the conjecture that STP behavior of synapses has the same functional dependence on *U* and τ_*rec*_, which allowed us to write a similar rule for the synaptic utilization *U*. Interestingly, such learning rules depend on the maximum synaptic strength, and they therefore: *(i)* provided a natural link between STP and STDP and *(ii)* allowed us to derive an STP-dependent rule for the maximum synaptic strength, to be added to the STDP contribution.

The interaction between short- and long-term plasticity is largely supported by experimental evidence (Markram et al., 1997), although the exact mechanisms are still unknown. Some results (Markram and Tsodyks, 1996; Sjöström et al., 2003, 2007) suggest that synapses become more/less depressing after long-term potentiation/depression. Our rules incorporate this behavior: long-term potentiation/depression always produces larger/smaller changes in STP parameters. However, whether these modifications bring more facilitation or depression critically depends on whether the population firing rate 〈ν〉 is approaching the target rate ν_*targ*_ from above or below. Consider, for example, Equation (16): if ν_*targ*_ − 〈ν〉 < 0, then long-term potentiation will produce a stronger depression, thus reproducing the experimentally observed behavior. In our simulations, this happens to the neurons that are firing at low frequencies. On the other hand, if ν_*targ*_ − 〈ν〉 > 0, then an increase in *A* will make τ_*rec*_ even smaller, resulting in a less depressing synapse. In our simulations, this happens to the neurons that are firing at high frequencies. A similar argument can be formulated for the induction of long-term depression. We note that several mechanisms have been identified to compete during synaptic transmission, resulting in a more complex and less clear relationship between STP and STDP (Sjöström et al., 2007).

In Sjöström et al. (2003, 2007) the authors link the interaction between short- and long-term plasticity with the frequency of firing: at high rates, synapses tend to become stronger and more depressing, while at lower frequencies they tend to become weaker and less depressing. Our derivation, instead, suggests the opposite: if we rely on the hypothesis that large values of τ_*rec*_ lead to depression and small values to facilitation (Chow et al., 2005), according to Equation (15), facilitating synapses allow neurons to reach higher frequencies. These findings, together with the STDP triplet rule, from the basis of our work: they provide the theoretical basis for the experimentally observed correspondence between facilitation and bidirectionality, and between depression and unidirectionality. The behavior expressed by Equation (15) is experimentally and computationally based on previous work that relates facilitation with high frequency and rate code, and depression with low frequency and temporal code (Fuhrmann et al., 2002; Blackman et al., 2013). This is because, for example, a facilitating synapse may require several spikes to elicit an action potential, meaning that only high frequency stimulation can generate postsynaptic spikes (Matveev and Wang, 2000; Klyachko and Stevens, 2006).

We derived our rules by minimizing an error function that is equal to zero when the target and actual firing rates are equal. Alternatively, we could have defined a reward function opposite to the error function in the sense that for zero error the reward function has its maximum value, and it is equal to zero for large error. We could have then taken the gradient of the reward function instead, bringing the derived rules into the framework of policy gradient learning methods and reinterpreting the feedback signal as a reward signal (Urbanczik and Senn, 2009; Vasilaki et al., 2009a; Richmond et al., 2011). In biological systems, dopamine is thought to act as reward signal (Schultz et al., 1997; Fiorillo et al., 2003), and its role in the context of learning associated with STDP, and more generally with Hebbian learning, has been extensively studied (Tobler et al., 2005; Izhikevich, 2007; Legenstein et al., 2008).

Each of the learning rules we proposed depends, however, on the difference between the target and the actual firing rates, computed at the population level. This implies the presence of: *(i)* a single feedback signal encoding the population activity, which is processed outside the population and broadcasted to all neurons; *(ii)* an external signal bringing information about the current paradigm, i.e., the target firing rate. Similar to Urbanczik and Senn (2009), we can assume that synapses receive both signals via ambient neurotransmitter concentrations, leading to an on-line plasticity rule.

We initially tested our learning scheme by implementing the rules for τ_*rec*_ and *U* on a classical paradigm of inverting associations: keeping the stimulus fixed and varying the associations, the network had to learn to first make choice A and then unlearn it in favor of choice B. This led to a network able to periodically switch its behavior from depressing to facilitating and vice versa, closely following the change in the association paradigm. Throughout the simulation, the network formed motifs similar to those experimentally observed in Wang et al. (2006) and Pignatelli (2009), with facilitating synapses developing bidirectional motifs and depressing synapses developing unidirectional motifs. The desirable motifs were formed due to two factors: (i) the triplet rule that governed long-term potentiation and (ii) the wave-like input stimulus of the network. The form of the plasticity rule guarantees that when neurons fire at high frequency, the synaptic efficacy increases. Hence, synapses will grow up to their bounds, leading to bidirectional connections. On the contrary, when neurons fire at low frequencies, the synaptic efficacy decreases, yet the wave-like input imposes unidirectional connectivity.

We further extended this learning model by adding an STP-motivated rule for the maximum synaptic strength, and we tested it on the same invert association scenario. Results showed the same behavior as before but with faster dynamics due to the joint action of STP and STDP on the absolute efficacy.

In the second part of the paper, we extended our study. First, we considered two populations that have to fire at different frequencies (low, high). Then, we introduced a learning rule for the facilitating time constant, in order to have a full learning model involving all four parameters. The aim was twofold:

*(i) Comparison of our results with experimental data in Wang et al. (2006)*. Although the accuracy is not excellent, we were able to qualitatively reproduce the basic differentiation in the ranges of values of the STP parameters, reflecting the existence of four different synaptic subtypes. We believe that by further adapting the model, in particular learning rates and target frequencies or by considering other rule combinations, it is possible to obtain different parameter values (in principle an infinite combination of them), and thus possibly reproduce the results of Wang and collaborators even better. However, we think this may not be critical because, as a recent study (Costa et al., 2013) has pointed out, fitting techniques generally used for deriving STP parameters from experimental data may give unreliable results. Given this limitation, we think it is important that our model accounts for a large variety of parameter values in principle, and that in this specific case of Wang et al. (2006) it is able to replicate the basic distinction in the synaptic response.

*(ii) Differentiation of synaptic types innervating two functionally different populations*. The reason for this lies in the way we constructed the learning model: what triggers the synaptic modification is the spike of the postsynaptic neuron. The firing rate of the population to which this postsynaptic neuron belongs is the information used to tune the values of STP parameters. In other words, we implement a target-specific learning mechanism. This choice is based on an optimization argument: the more direct and efficient way for a neuron to influence its own activity through synaptic changes is to modify incoming synapses rather than outgoing synapses. A second scheme, a source-specific learning mechanism modifying the outgoing synapses, would have probably led to the same results within closed microcircuits, but on a much longer time scale.

Our target-specific learning mechanism is also supported by experimental evidence (see Blackman et al., 2013 for a review). Despite the fact that STP seems to be mainly a presynaptic mechanism, it has been shown that the target cells can also determine the STP dynamics. All the studies we are aware of have established such a target specificity only in the context of excitatory cells innervating other excitatory cells on one hand and inhibitory cells on the other, specially interneurons (Markram et al., 1998b; Reyes et al., 1998; Buchanan et al., 2012). It would therefore be interesting to appropriately modify the double population scenario by incorporating a population of inhibitory neurons and comparing the results with existing data. In addition, some authors (Blackman et al., 2013; Costa et al., 2013) suggested that a similar differentiation might exist within excitatory only populations. Having target-specific STP for excitatory-excitatory connections is still an open possibility that needs to be further explored. Here we show from a theoretical point of view that such a differentiation is possible between fundamentally similar (all excitatory) but functionally different (encoding for different paradigms) targets.

The well-established existence of STP-target specificity provides us with a possible biological explanation for the learning rules we derived. Indeed, this scenario requires that the postsynaptic neuron can regulate specifically its own presynaptic compartment only, by a retrograde signal that does not affect neighboring cells. Thus, diffusive retrograde messengers, for example endocannabinoids and nitric oxide, do not appear to be the most suited agents, whereas synaptic adhesion molecules, for example cadherins (Bozdagi et al., 2004) and neuroligins (Dean and Dresbach, 2006), seem to be better candidates for playing this role. These molecules are responsible for governing the presynaptic transmitter release through many different presynaptic mechanisms (Zucker and Regehr, 2002; Blatow et al., 2003; Deng et al., 2011; Blackman et al., 2013).

We underline that the way we obtained the learning rules is based in part on heuristic evaluation. According to Equation (15), derived from a semi-rigorous argument, the key parameters seems to be τ_*rec*_ and *A*. By also including *U* following Carvalho and Buonomano (2011), we obtain a learning scheme involving τ_*rec*_, *U* and *A* only, which we used to study the double population problem and evaluate the importance of τ_*facil*_. Results remain essentially unchanged from the full model, suggesting that τ_*facil*_ does not play a critical role in the task we defined. This is not surprising and the reason is that our rules link facilitation with a high firing rate, and depression with a low firing rate. Indeed, even with a small facilitation time constant (small τ_*facil*_), synapses are still able to fire at a high rate, as long as the stimulating frequency is high enough and recovery from depression is fast enough (low τ_*rec*_). Therefore, the time constant of recovery from depression seems to be the only parameter regulating the firing frequency of the neuron for high firing rates, exactly as it comes out from the objective function (we recall that Equation 15 comes from an inequality obtained in the limit of high frequency). With our novel view of allowing synapses to modify their properties from facilitating to depressing and vice versa, we therefore suggest that τ_*rec*_ is the parameter that is mostly related to rate coding, whereas *U* to temporal coding.

This conclusion is also supported by Carvalho and Buonomano (2011). In this paper the authors described a simple problem based on temporal synchrony between two inputs that cannot be solved unless STP is learnt, together with STDP. Besides the long-lasting change in *A*, they introduce a temporal synaptic plasticity for *U* only and they showed that this indeed solves the problem. Also, they reported that changing *U* only was the most efficient way to solve the problem. Our work supports the hypothesis that, when dealing with rate coding tasks, the only necessary parameter that has to be learnt is τ_*rec*_, whereas, based on Carvalho and Buonomano (2011), when dealing with temporal coding tasks, the only necessary parameter is *U*.

Another result pointing to a similar direction can be found in Natschläger et al. (2001), where the authors use optimization techniques, rather than explicit learning rules, to train a network of neurons in order to transform a time-varying input into a desired time-varying output. They show that to achieve good performance, one needs to change at least two parameters, either *A* and τ_*rec*_, or *A* and *U*. This confirms that learning must involve at least one presynaptic and one postsynaptic parameter, and that τ_*facil*_ seems not to be relevant in these types of tasks.

We finally presented results from what we call the minimal model, where only τ_*rec*_ and *A* were allowed to change, since both their corresponding update rules come directly from the gradient of the objective function we defined. Results confirmed our belief, as we were still able to learn the tasks while obtaining results similar to those from Wang and collaborators. It is in agreement with our conjecture that when we tried to apply learning on *U* and *A* only (results now shown here), the network failed to perform its task because the population that was supposed to fire high stabilized at a much lower frequency, i.e., ~ 15 Hz. Therefore, an alternative minimal model adapting *U* and *A* would be able to successfully learn only targets of a lower firing regime. We believe that specialization of parameters in the STP model depending on tasks and signal encoding may be a key ingredient toward a better understanding of synaptic and neuron functionality.

## Author contributions

All authors provided materials and contributed to writing the article.

### Conflict of interest statement

The authors declare that the research was conducted in the absence of any commercial or financial relationships that could be construed as a potential conflict of interest.
